# Study on the Antitumor Mechanism of Tanshinone IIA In Vivo and In Vitro through the Regulation of PERK-ATF4-HSPA5 Pathway-Mediated Ferroptosis

**DOI:** 10.3390/molecules29071557

**Published:** 2024-03-30

**Authors:** Chunxiang Guo, Wei Zhao, Wei Wang, Zheng Yao, Wenhui Chen, Xiaoyi Feng

**Affiliations:** 1 School of Basic Medicine, Yunnan University of Chinese Medicine, Kunming 650500, China; gcx411361727@126.com (C.G.); weifry.cool.0606@163.com (W.Z.); ivan_ww@163.com (W.W.); yaozhmail@126.com (Z.Y.); 2 Yunnan Provincial Key Laboratory of Molecular Biology for Sinomedicine, Kunming 650500, China

**Keywords:** Tanshinone IIA, endoplasmic reticulum stress, PERK-ATF4-HSPA5 pathway, ferroptosis, anti-hepatoma

## Abstract

As a traditional Chinese medicine, *Salvia miltiorrhiza Bunge* was first recorded in the Shennong Materia Medica Classic and is widely used to treat “the accumulation of symptoms and masses”. The main active ingredient of *Salvia miltiorrhiza Bunge*, Tanshinone IIA (TIIA), has shown anti-inflammatory, antitumor, antifibrosis, antibacterial, and antioxidative activities, etc. In this study, the results showed that TIIA could inhibit the proliferation and migration of HepG2 cells and downregulate glutathione (GSH) and Glutathione Peroxidase 4 (GPX4) levels; besides, TIIA induced the production of Reactive Oxygen Species (ROS), and upregulated the total iron content. Based on network pharmacology analysis, the antitumor effect of TIIA was found to be focused on the endoplasmic reticulum (ER)-mediated ferroptosis signaling pathway, with protein kinase R (PKR)-like ER kinase (PERK)-activating transcription factor 4 (ATF4)-heat shock 70 kDa protein 5 (HSPA5) as the main pathway. Herein, TIIA showed typical ferroptosis characteristics, and a ferroptosis inhibitor (ferrostatin-1) was used to verify the effect. The antitumor effects of TIIA, occurring through the inhibition of the PERK-ATF4-HSPA5 pathway, were further observed in vivo as significantly inhibited tumor growth and the improved pathological morphology of tumor tissue in H22-bearing mice. In summary, the antitumor mechanism of TIIA might be related to the downregulation of the activation of PERK-ATF4-HSPA5 pathway-mediated ferroptosis.

## 1. Introduction

Hepatocellular carcinoma (HCC) accounts for nearly 90% of primary liver cancer, which is the main global public health burden [1,2]. Non-alcoholic steatohepatitis (NASH), alcoholic steatohepatitis, viral hepatitis, and chronic hepatic injury are the main risk factors for HCC [3,4]. These pathogenic factors induce the occurrence of HCC by promoting oxidative stress, inflammation, and gene mutations, which accelerate the progression of the endoplasmic reticulum (ER) towards a state of stress [5]. ER stress (ERS) and its mediated cell apoptosis are closely related to the occurrence and development of HCC [6,7].

When ERS occurs, protein kinase R (PKR)-like ER kinase (PERK) is activated, further phosphorylating eukaryotic initiation factor 2α (eIF2α), which selectively increases the translation of the activating transcription factor (ATF4) [8,9]; then, heat shock 70 kDa protein 5 (HSPA5) transcription can be activated [10]. Recent studies have shown that the activation of the ATF4-HSPA5 pathway is a stress-adaptation response against ferroptosis [11]. Further, the overexpression of HSPA5 could inhibit glutathione peroxidase 4 (GPX4) degradation to reduce lipid peroxidation in ferroptosis [11]. Therefore, the signaling pathway of PERK-ATF4-HSPA5 is closely related to the occurrence of ferroptosis.

*Salvia miltiorrhiza Bunge* was first recorded in the “Shen nong Materia Medica Classic”. It reportedly had the functions of promoting blood circulation to remove blood stasis, nourishing blood to tranquillize the mind, and cooling blood to disperse carbuncles [12]. Tanshinone IIA (TIIA) is a phenanthraquinone compound extracted from *Salvia miltiorrhiza Bunge*, as the main bioactive ingredient, which has anti-inflammatory [13,14], antiatherosclerotic [15,16], antitumor [17,18], antifibrosis [19], antibacterial [20], and antioxidative properties [21,22], and other pharmacological effects. Previous studies have shown that TIIA can promote tumor cell apoptosis by increasing PERK, ATF6, and IRE1α levels in pancreatic cancer xenograft tumors [23]. However, researchers have reported that TIIA can suppresses ERS-induced apoptosis [24,25], and can induce ferroptosis in tumor cells [26]. As we know, there is a crosstalk between ERS and ferroptosis [27], and the effect of TIIA in hepatoma cells and the potential regulatory mechanisms remain unclear. Therefore, the study aimed to explore the possible anti-HCC mechanism of TIIA and provide new therapeutic directions for HCC.

## 2. Results

### 2.1. Tanshinone IIA Inhibited the Proliferation and Migration of HepG2 Cells

**Figure 1:**
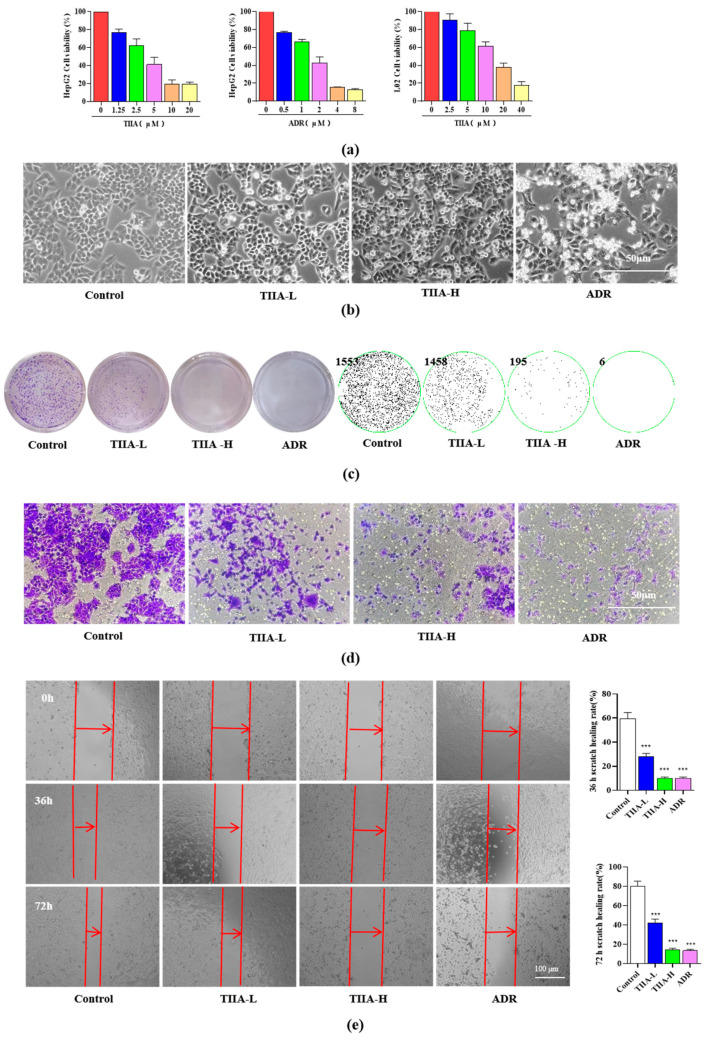
Tanshinone IIA inhibited the proliferation and migration of HepG2 cells. TIIA-L, low concentration of TIIA (2.5 μM); TIIA-H, high concentration of TIIA (10 μM); ADR (2 μM). (**a**) HepG2 cells were treated with different concentrations of TIIA (IC_50_ = 4.17 ± 0.27 μM), ADR (IC_50_ = 1.45 ± 0.10 μM); L02 cells were treated with different concentrations of TIIA (IC_50_ = 13.55 ± 1.32 μM). (**b**) The morphology of HepG2 cells treated with TIIA for 24 h. (**c**) The colony tests of HepG2 cells with TIIA treatments was conducted for 7 days. (**d**) The transwell of HepG2 cells treated with TIIA for 48 h. (**e**) The scratch experiments of HepG2 cells treated with TIIA for different time periods (0, 36, and 72 h). The red arrow indicates the distance of the scratch. *** *p* < 0.001 vs. control group.

TIIA inhibited the cell viability of HepG2 cells; the half-maximal inhibitory concentration (IC_50_) of TIIA was 4.17 ± 0.27 μM. Adriamycin (ADR) was used as a positive control, and the IC_50_ was 1.45 ± 0.10 μM. Meanwhile, TIIA inhibited the cell viability of L02 cells, and the IC_50_ was 13.55 ± 1.32 μM. Compared with the IC_50_ of TIIA on HepG2 cells (4.17 ± 0.27 μM), the results showed a significant difference. Therefore, we used the two concentrations to discuss the antitumor effect; they were approximately half of the IC_50_ value (2.5 μM) and double it (10 μM) in HepG2 cells. And the two concentrations were lower than the half lethal concentration of L02 cells (Figure 1a). TIIA could significantly change the morphology of HepG2 cells, with a significant decrease in cell density, sparse growth, cell shrinkage and detachment, and a relative increase in suspended cells (Figure 1b). In addition, TIIA inhibited the colony formation and the migration of HepG2 cells in a dose-dependent manner (Figure 1c–e). These results showed that TIIA could induce cell death in HepG2 cells.

### 2.2. Analysis of the Mechanisms and Molecular Targets of TIIA Treatment of Hepatocellular Carcinoma

**Figure 2:**
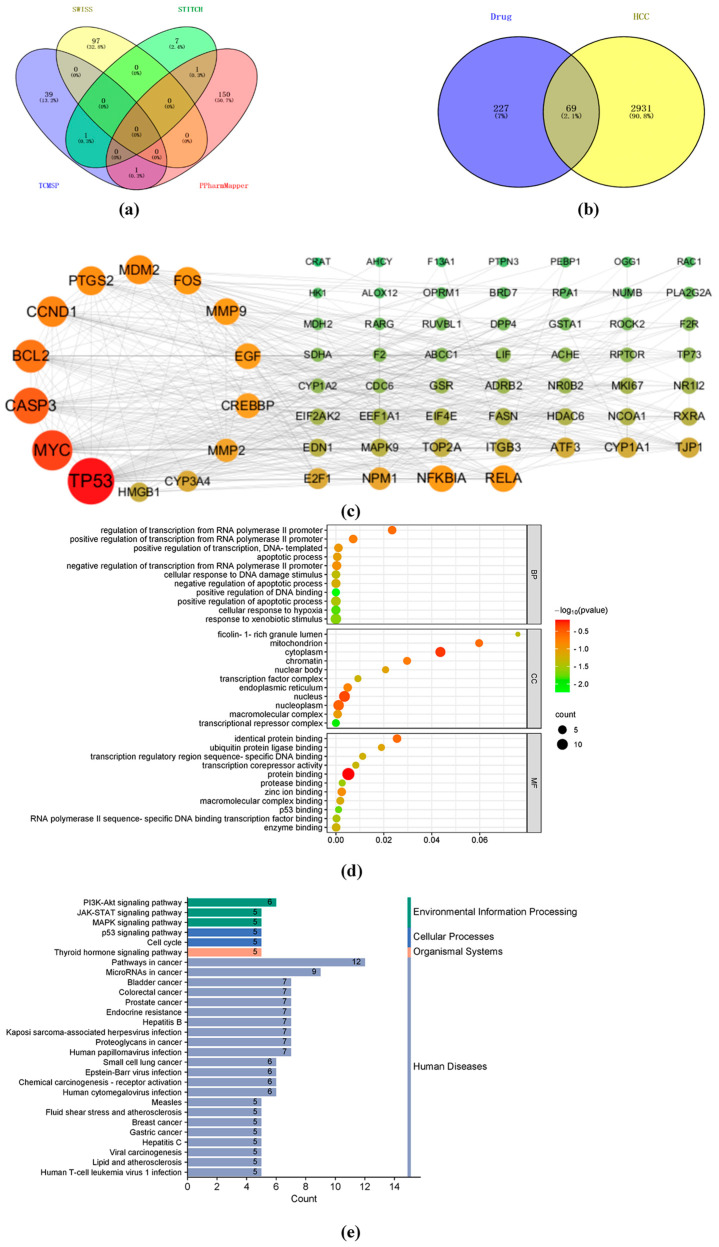
Compound target network of TIIA and signal pathway enrichment. Using the Venny online tool, (**a**) 296 targets of TIIA and (**b**) 69 targets of TIIA and hepatocellular carcinoma were screened out. (**c**) The size and color of the circle are arranged according to the degree of correlation, with the 14 most likely targets forming a ring on the left side. Circles that are darker in color and larger circles indicate a higher correlation. (**d**) GO functional enrichment analysis and (**e**) KEGG enrichment analysis based on the DAVID database.

Based on the antitumor effects of TIIA, the putative antitumor pathways of TIIA were predicted by Network pharmacology. A total of 296 possible targets of TIIA were obtained from four databases, namely 41 from the TCMSP database, 9 from the STITCH database, 152 from the PharmMapper database, and 97 from the SwissTargetPrediction database (Figure 2a). And 3000 tumor-related intersection targets were screened out through the Genecards database. The Venn diagram shows 69 main targets of liver cancer (Figure 2b) from 3000 common targets. We can visualize the targets of liver cancer in the form of a drug target disease network using Cytoscape v. 3.9.1 software. The 69 common targets are represented in different colors according to the degree (>12.30), closeness (>0.008) and betweenness (>66.54), and the 14 most likely tumor-related targets from the screening are shown in Figure 2c. The GO functional enrichment analysis and KEGG pathway enrichment analysis are based on the DAVID database. The functional enrichment of GO involves such functions as protein binding, ubiquitin protein ligase binding, etc. Regarding the site, mainly the nucleus, nucleoplasm, and endoplasmic reticulum are included. Molecular functions related to endoplasmic reticulum protein folding, including DNA transcription factor activity and characteristic RNA polymerase II activity regulation, were observed (Figure 2d). Pathways from KEGG enrichment include the phosphatidylinositol 3-kinase (PI3K)-AKT pathway, the Janus kinase (JAK) signal transducer and activator of the transcription (STAT) pathway, and the mitogen-activated protein kinase (MAPK) pathway, among others. Those were mainly concentrated in the positive regulation of protein kinase B signaling in the cytoplasm, involving the nucleus, including the endoplasmic reticulum lumen (Figure 2e). Otherwise, these signaling pathways usually contributed to apoptotic signaling through the unfolded protein reaction (UPR) [28,29,30]. Based on this comprehensive analysis, it appears that the antitumor effect of TIIA may be related to upstream ER signal transduction. We suggested that the ER-mediated PERK-ATF4 signaling pathway was mainly involved in the procedure.

### 2.3. Tanshinone IIA-Promoted Ferroptosis of HepG2 Cells

**Figure 3:**
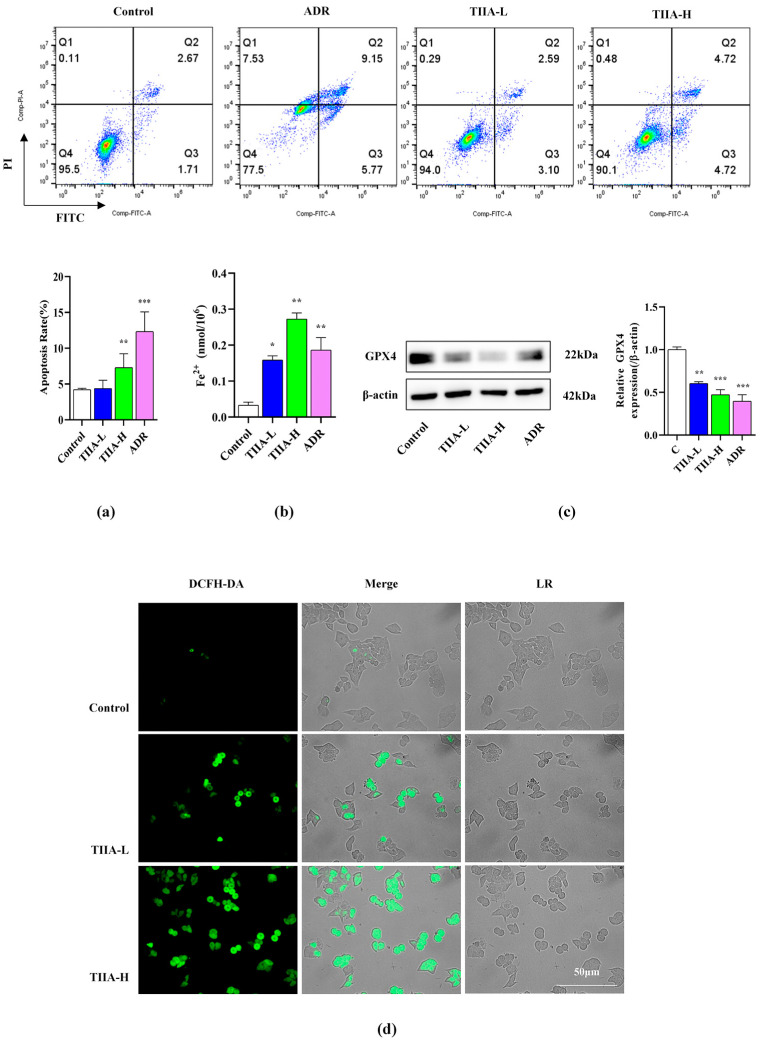
TIIA promoted ferroptosis of HepG2 cells. TIIA-L, low concentration of TIIA (2.5 μM); TIIA-H, high concentration of TIIA (10 μM); ADR (2 μM). HepG2 cells treated with TIIA for 24 h. (**a**) Flow cytometry assay double-stained with Annexin V and PI. Histogram statistics of cell death. The number of cell clusters increases, and the image color changes from blue to red. (**b**) Determination of the total iron content. (**c**) The expression of GPX4 was detected using Western blot. (**d**) Fluorescence staining of ROS in HepG2 cells. Green fluorescence represents the production of ROS. Data were presented as mean ± SD. * *p* < 0.05, ** *p* < 0.01, and *** *p* < 0.001 vs. control group.

TIIA significantly induced nuclear and cell membrane morphological changes in HepG2 cells, as shown through Annexin V/PI staining, at as low as 2.5 µmol·L^−1^ (Figure 3a). Compared with the control group, TIIA significantly increased the content of Fe^2+^, according to the ELISA test (Figure 3b). TIIA downregulated the expression of GPX4 protein (Figure 3c). In addition, DCFH-DA staining also showed that TIIA significantly increased ROS levels, which thus promoted ferroptosis (Figure 3d).

### 2.4. Inhibition of the Activation of the PERK-ATF4-HSPA5 Signaling Pathway in HepG2 Cells

**Figure 4:**
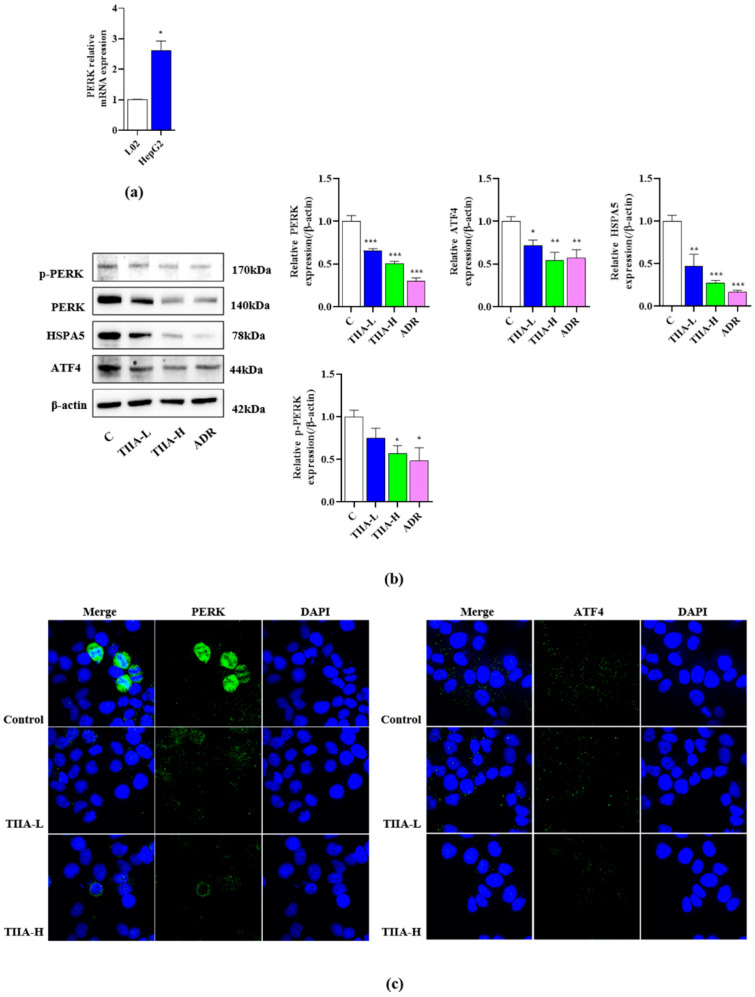
TIIA inhibited the PERK-ATF4-HSPA5 signaling pathway. TIIA-L, low concentration of TIIA (2.5 μM); TIIA-H, high concentration of TIIA (10 μM); ADR (2 μM). HepG2 cells treated with TIIA for 24 h. (**a**) Expression levels of PERK mRNA. (**b**) The expressions of PERK, ATF4, HSPA5, GPX4, and p-PERK were measured using Western blot. (**c**) The fluorescence image of the laser confocal detection of the expression of PERK (green) on the HepG2 cell membrane and ATF4 (green) in HepG2 cells (10 × 60). Blue pseudocolor is fluorescent DNA dye (DAPI). Scale bar = 50 μm. * *p* < 0.05, ** *p* < 0.01, and *** *p* < 0.001 vs. control group.

Based on the effect of TIIA to promote ferroptosis, and to further trace PERK and ATF4 changes in HepG2 cells treated with TIIA, we detected the effect of TIIA on the activation of the PERK signaling pathway. Firstly, mRNA analysis revealed higher levels of PERK in HepG2 cells compared to L02 cells (Figure 4a). Compared with the control group, TIIA significantly decreased the expression levels of PERK, ATF4, HSPA5, and p-PERK in a dose-dependent manner (Figure 4b). In addition, the fluorescence intensity of PERK and ATF4 was detected through fluorescence staining. The result showed that TIIA decreased the levels of PERK and ATF4 protein (Figure 4c).

**Figure 5:**
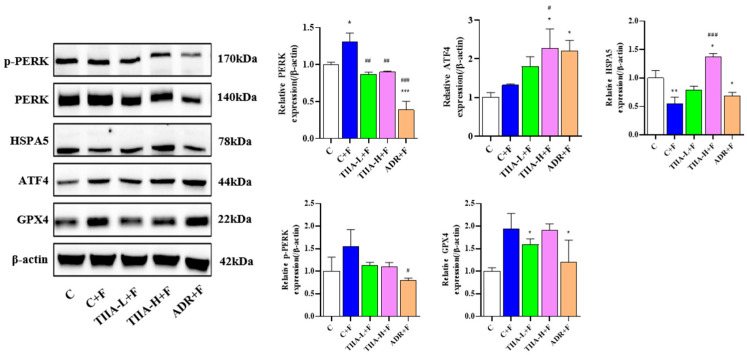
Ferrostatin-1 blocked the antitumor effect of TIIA. TIIA-L, low concentration of TIIA (2.5 μM); TIIA-H, high concentration of TIIA (10 μM); ADR (2 μM). PERK, ATF4, HSPA5, GPX4, and p-PERK expression in HepG2 cells measured by Western blotting after ferrostatin-1 was added for 1 h, the culture medium containing ferrostatin-1 was discarded, and treatment took place with TIIA for 24 h. Data are presented as mean ± SD.* *p* < 0.05, ** *p* < 0.01, and *** *p* < 0.001 vs. C group. ^#^ *p* < 0.05, ^##^ *p* < 0.01, and ^###^ *p* < 0.001 vs. C + F group.

In order to further study the effect of TIIA, ferrostatin-1 (ferroptosis inhibitor) was used to verify the effect of TIIA. Compared with the inhibitor group (C + F), the effects of TIIA on the expression of ATF4, HSPA5, and GPX4 were reversed (Figure 5). The results show that the effect of TIIA-mediated cell ferroptosis is blocked by ferrostatin-1. To summarize, the results suggested that TIIA exerted an anti-HepG2 effect by inhibiting the PERK-ATF4-HSPA5 pathway and inducing ferroptosis in HepG2 cells.

### 2.5. Docking and Cellular Thermal Shift Assay

**Figure 6:**
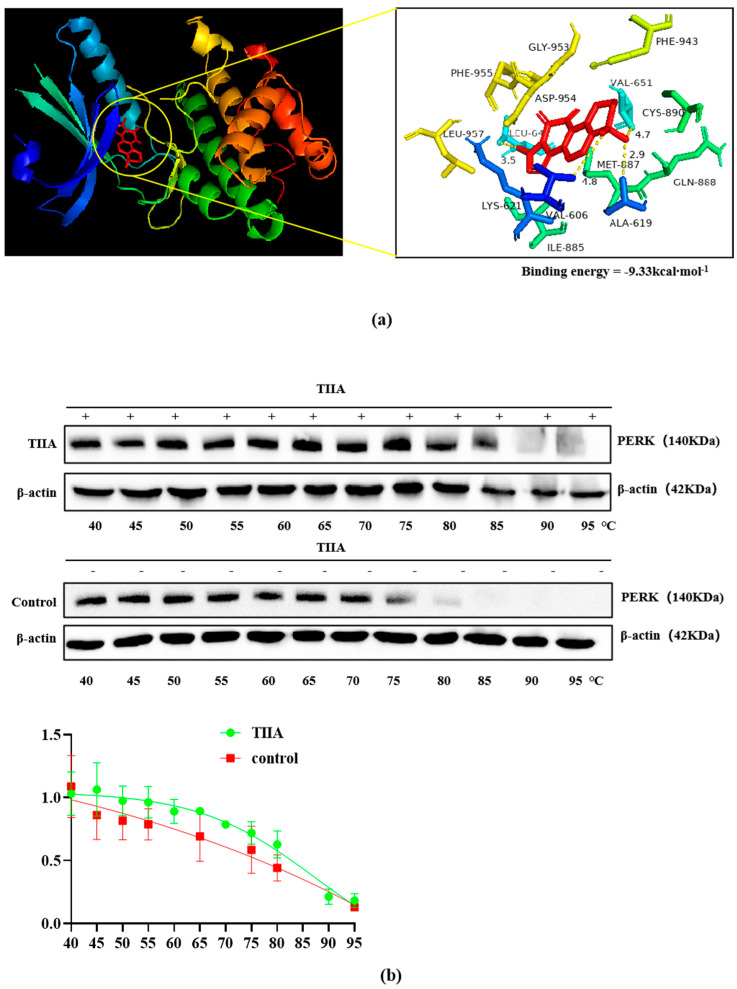
TIIA binds to PERK and increases the denaturation temperature. HepG2 cells were treated with 10 μM TIIA for 1 h. (**a**) The structure complex of PERK-TIIA. TIIA is depicted in red, and the chains of PERK subunits are represented by green, cyan blue, and magenta. Data deposition: the crystallography, atomic coordinates, and structural factors were deposited in the Protein Data Bank, www.pdb.org (accessed on 14 December 2023) (PDB ID code 4G34). (**b**) The CETSA binding assay of PERK and β-actin in the presence or absence of TIIA (20 µM) at different temperatures was detected using Western blot. The temperature-dependent melting curves and the apparent aggregation temperature were calculated by nonlinear regression. Values represent the mean ± SD (*N* = 3 replicates).

To determine whether TIIA could bind to the active pocket of PERK (PDB code 4G34), we predicted its binding ability with the use of AutoDock4 software (1.5.6 Sep_17_14). We found that TIIA mainly interacts with residues involved in PERK activation, such as LEU, LYS, PHE, ALA, VAL, ASP, GLY, and ILE. The main forces of their interactions were determined to be the conjugation effect and hydrogen bonding force (Figure 6a). The binding energy was calculated as −9.33 kcal·moL^−1^. Herein, the cellular thermal shift assay (CETSA) was used to verify the capability of TIIA to bind to PERK. The result showed that the target protein was degraded as the temperature increased, but the denaturation temperature of the combination drug group was higher than that of the control group (Figure 6b).

### 2.6. Tanshinone IIA Inhibited Tumor Growth in H22 Tumor-Bearing Mice

**Figure 7:**
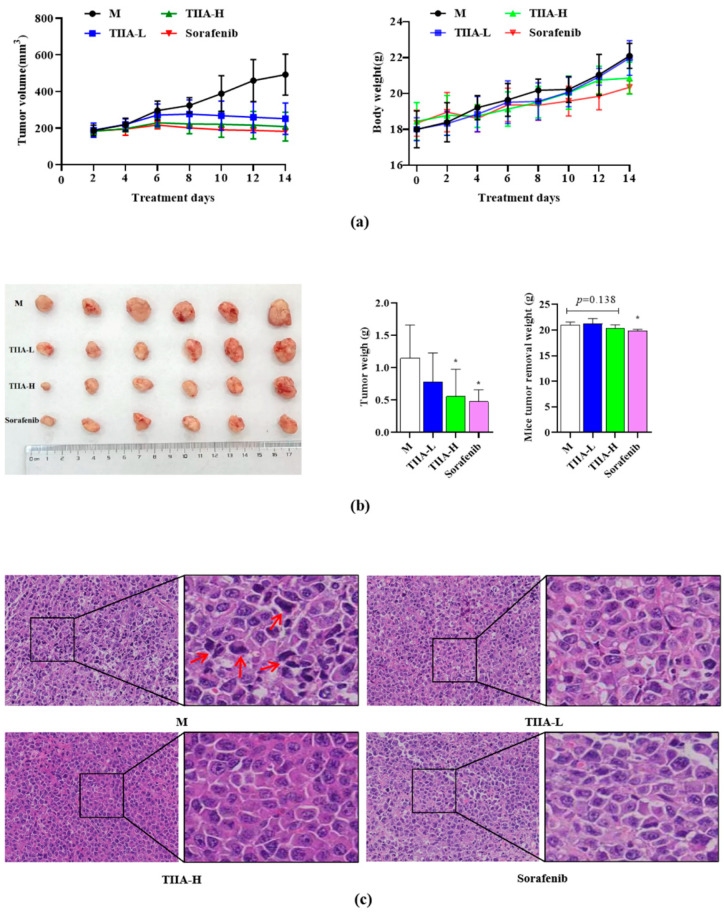
TIIA-inhibited tumor growth in H22 mice. M: saline model group, TIIA-L: low dosage of TIIA (20 mg/kg), TIIA-H: high dosage of TIIA (50 mg/kg), Sorafenib (60 mg/kg). (**a**) The tumor volume and body weight were calculated every two days. (**b**) The tumor removal weight. (**c**) The morphology of tumors was assayed with H&E staining. The red arrow indicates that multi-level and asymmetric division phenomena can be observed in the tumor cells of the model group. * *p* < 0.05 vs. model group.

Based on the previous results, we further determined whether the effect of TIIA was consistent in vivo. Compared with the model group, all drug groups were able to effectively inhibit the tumor volume growth, especially in the sorafenib group (*p* < 0.05). And we found that the weight of H22 tumor-bearing mice in each group showed a gradually increasing trend, and there was no significant difference between the different groups (Figure 7a); however, the tumor removal weight was obviously different, and the tumor weights of the drug groups were decreased (Figure 7b). The tumor nuclei exhibited heterogeneous morphology and were deeply stained, and multilevel and asymmetric division phenomena were visible (red arrow) in the model group. The tumor cells in the drug group were tightly connected, and the number of nuclear abnormalities was significantly reduced, especially in the TIIA group (Figure 7c).

### 2.7. Tanshinone IIA Inhibited the PERK-ATF4-HSPA5 Signaling Pathway In Vivo

**Figure 8:**
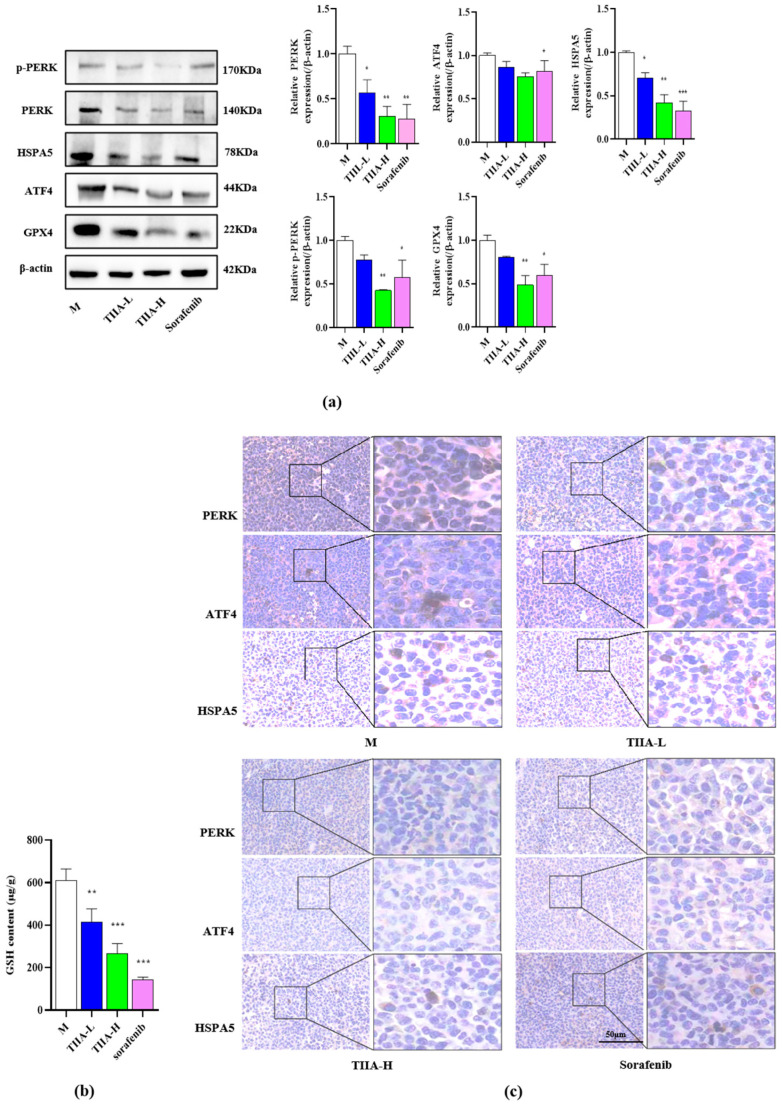
TIIA inhibited the PERK-ATF4-HSPA5 signaling pathway and decreased the GPX4 level in tumor tissue. M: saline model group, TIIA-L: low dosage of TIIA (20 mg/kg), TIIA-H: high dosage of TIIA (50 mg/kg), Sorafenib (60 mg/kg). (**a**) The expressions of PERK, ATF4, HSPA5, GPX4, and p-PERK were detected using Western blot. (**b**) GSH level of tumor tissue. (**c**) The immunohistochemistry was analyzed. * *p* < 0.05, ** *p* < 0.01, and *** *p* < 0.001 vs. model group.

Compared with the model group, TIIA inhibited the expression of PERK, ATF4, HSPA5, GPX4, and p-PERK in a dose-dependent manner (Figure 8a). In addition, TIIA decreased the GSH level in tumor tissues (Figure 8b). The immunohistochemistry results were consistent with the Western blot result (Figure 8c).

## 3. Discussion

The liver, as one of the main organs involved in metabolic diseases, is the main organ for lipid metabolism [31]. And ERS is closely related to the development of metabolic liver diseases [32,33]. Usually, tumor cells maintain the ERS at a high level to protect themselves from internal and external stimuli, such as hypoxia, ischemia, excessive proliferation, blocked apoptosis, and starvation [34]. Excessive and intense ERS induces the occurrence of HCC, which is one of the key factors in the progression of NASH to HCC [35]. However, the promotion or inhibition of ERS can promote the apoptosis of tumor cells and exert anti-tumor effects [8]. Under ERS, the activation of PERK-ATF4 signaling can upregulate the expression of HSPA5. HSP5A inhibits the degradation of GPX4 and inhibits ferroptosis [36]. As reported in the literature, there is crosstalk between ERS and ferroptosis [27]. Ferroptosis can activate ERS and the activation of ERS pathways inhibits ferroptosis, leading to drug resistance in cancer. Therefore, elucidating the relationship between ERS and ferroptosis in response to TIIA treatment has great significance.

In recent years, the anti-tumor research of TIIA has become a hot topic, and there have been many reports on its anti-tumor effects. The reported mechanisms include the inhibition of the PI3K-AKT signaling pathway [37], the Akt/WEE1/CDK1 signaling pathway [38], the EGFR signaling pathway [39], and the ATF4-JAK signaling pathway [40], etc. But the antitumor mechanisms of TIIA have not been illuminated, or whether TIIA has the ability to regulate ERS and ferroptosis, thereby promoting cell apoptosis and inhibiting tumor growth. Therefore, this study aimed to explore the possible anti-HCC mechanism of TIIA.

In the present study, we found that TIIA could inhibit the proliferation and migration of HepG2 cells, promote morphological changes, induce mitochondrial dysfunction and ROS accumulation, and change the cell membrane morphologically. In order to elucidate its anti-HepG2 molecular mechanism, the putative antitumor pathways of TIIA were predicted using Network pharmacology analysis. According to the degree (>12.30), closeness (>0.008), and betweenness (>66.54), the 14 most-tumor-related targets were screened from 69 intersection targets. The antitumor effect of TIIA was found to be focused on anticancer signaling pathways. Combined with the results of TIIA on HepG2 cells, we suggest that the potential mechanism involves the ER-mediated ferroptosis signaling pathway, and that PERK-ATF4-HSPA5 is the main signaling pathway involved in this process. PERK-ATF4 signaling is the main pathway by which the homeostasis of protein synthesis is maintained during ERS. Under ERS, the activation of PERK leads to the phosphorylation of the α-subunit of eukaryotic initiation factor 2 (eIF2α) and ATF4. On the other hand, ATF4 promotes the expression of pro-survival genes related to protein folding [41]. Meanwhile, the activation of PERK-ATF4 signaling can upregulate the expression of HSPA5, which inhibits the degradation of GPX4. Therefore, the inhibition of the PERK-ATF4 pathway promotes cell ferroptosis by downregulating GPX4 expression and increasing both Fe^2+^ and ROS levels. In this study, we observed, using HepG2 cells, that TIIA not only inhibits the activity but also decreases the levels of PERK, with the subsequent regulation of the ATF4 and HSPA5 signaling pathways downstream, thereby resulting in suppressed levels of GPX4 and increased concentrations of Fe^2+^ in promoting ferroptosis. We suggest that TIIA induced the downregulation of PERK, thereby inhibiting the PERK-ATF4-HSPA5 signaling pathways and exerting its anti-HepG2 effect. The ferroptosis inhibitor (ferrostatin-1) was used to verify the effect of TIIA. Compared with the control group, the downregulatory effect of TIIA on PERK-ATF4-HSPA5 was blocked by the ferroptosis inhibitor. 

Further study of TIIA on the H22 tumor-bearing mice model was conducted. The results showed consistency with the in vitro experiments. TIIA could significantly reduce the levels of GPX4 and GSH in tumor tissues, and inhibited tumor growth through the downregulation of the PERK-ATF4-HSPA5 signaling pathway. These data indicate that ferroptosis plays a key role in TIIA-induced hepatocellular carcinoma cell death.

## 4. Materials and Methods

### 4.1. Reagents

TIIA was purchased from Sigma, Saint Louis, MO, USA (Lot: T4952-25MG, purity ≥ 97%). Fetal Bovine Serum (FBS) was purchased from CORNING, Corning, NY, USA (Lot: 19621001). In addition, 0.25% Trypsin-EDTA, Dulbecco’s Modified Eagle Medium (DMEM), and RPMI Medium 1640 basic were purchased from Gibco, Billings, MT, USA (Lot: 2509042, 8123473, 8122237). Streptomycin–penicillin, tris-buffered saline (TBST), phosphate-buffered saline (PBS), 4% paraformaldehyde, and 0.1% Crystal Violet Staining Solution were purchased from Biosharp, Hefei, China (Lot: 21036205, 2311613, 23257166, 22360481, BL802A). Sorafenib, RIPA lysis buffer (strong), and the Annexin V-FITC cell apoptosis detection kit were purchased from Beyotime, Nantong, China (Lot: 082622221109, P0013B, 122221220825). Dimethyl sulfoxide (DMSO) was purchased from Sigma (Lot: RNBK4363). Furthermore, the 0.9% sodium chloride injection was purchased from Kunming Nanjiang Pharmaceutical Co., Ltd., Kunming, China, and the batch number was C22083001. ATF4 was obtained from AffinitY, San Francisco, CA, USA (Lot: 39q6689). PERK, HSPA5, and p-PERK were purchased from CST, Danvers, MA, USA (Lot: D11A8, C50B12, 16F8). TRIZOL reagent was purchased from Ambion, Austin, TX, USA (LOT: 400208). Rever Tra Ace^TM^ qPCR RT Master Mix with gDNA Remover and SYBR Green Realtime PCR Master Mix were purchased from TOYOBO, Tokyo, Japan (LOT: 238000, 241000). BDS-PAGE loading buffer, 5×, the reactive oxygen species (ROS) assay kit, and glutathione (GSH) were purchased from Solarbio, Beijing, China (Lot: 20230415, 20230317, 2304001). Ferrostatin-1 was purchased from MedChemExpress, Monmouth Junction, NJ, USA (LOT: 149105). Biochemical Assay Reagent was purchased from Elabscience, Wuhan, China (LOT: DP02LT485470). 

### 4.2. Network Pharmacology

The potential targets of TIIA came from 5 databases, including Swiss Target Prediction (http://www.swisstargetprediction.ch/result.php?job=1138325903&organism=Homo_sapiens, accessed on 12 December 2023), TCMSP databases (https://old.tcmsp-e.com/molecule.php?qn=7154, accessed on 12 December 2023), STITCH databases (http://stitch.embl.de/cgi/network.pl?taskId=FcvjOB40Dmuk, accessed on 12 December 2023), PharmMapper databases (http://lilab-ecust.cn/pharmmapper/submitjob.html, accessed on 12 December 2023), and Genecards databases (https://www.genecards.org/Search/Keyword?queryString=Hepatocellular%20carcinoma, accessed on 12 December 2023). Possible targets of liver cancer were obtained from GeneCards database. The Venn diagram was drawn using the online platform (https://bioinfogp.cnb.csic.es/tools/venny/, accessed on 12 December 2023). The intersection targets were screened out through the String online database (https://cn.string-db.org/cgi/network?taskId=bWkww4EgyZUe&sessionId=bC2EjEIU2hMR, accessed on 12 December 2023). Further analysis was conducted on the relationship between the components and targets of TIIA, and a component target interaction network was constructed. The degree, betweenness centrality, and closeness centrality of the network topology feature values were analyzed with Cytoscape 3.9.1, and the most likely tumor-related targets selected from the candidate targets. By comprehensively analyzing the Term P Value and degree values, it was found that tumor-related signaling pathways are the likely targets of chemical components involved in tumor-related signaling pathways (http://www.bioinformatics.com.cn/plot_basic_pathway_enrichment_categorical_bar_plot_124, accessed on 12 December 2023).

### 4.3. Cells and Animal Model

The HepG2 cells and L02 cells came from preserved cells from the laboratory of the Liver Disease Research Group of Yunnan University of Chinese Medicine. H22 cells were purchased from Shanghai Saibaikang. HepG2 cells were incubated in DMEM with 10% FBS and 1% streptomycin–penicillin in a 5% CO_2_ chamber at 37 °C and 95% humidity. H22 cells and L02 cells were incubated in 1640 with 10% FBS. The morphology of the cells was observed using an inverted microscope at ×100 magnifications.

C57BL/6 mice (male, body weight 18 ± 2 g, and aged 6–7 w) were purchased from Sibeifu (Beijing) Biotechnology Co., Ltd., Beijing, China (SCXK (Beijing) 2019-0010). The animal batch number was 110324231106255318. The animal ethics approval number was R-062020G076, R-0620225002 from the Animal Experiment Ethics Review Committee of Yunnan University of Chinese Medicine. The mice were subjected to adaptive feeding for one week. Then, the mice were subcutaneously injected with H22 cells (2 × 10^6^ cells/0.2 mL). After 5~7 days, when the tumor volumes reached 50~100 mm^3^, the mice were randomly divided into 4 groups and treated with normal saline, sorafenib (60 mg/kg), a low dosage of TIIA (20 mg/kg), and a high dosage of TIIA (50 mg/kg) through intragastric administration once per day for 14 days. The model group was given the same volume of normal saline. The weight and tumor volumes of mice were measured once every 2 days. Tumor volumes = (A × B^2^)/2 (A: the tumor length and B: the tumor width). Tumor inhibition rate = (${\overset{-}{X}}_{\mathrm{model}\mathrm{group}}$ − ${\overset{-}{X}}_{\mathrm{drug}\mathrm{group}}$)/${\overset{-}{X}}_{\mathrm{model}\mathrm{group}}$. 

### 4.4. Cell Viability Assay 

HepG2 cells (1 × 10^4^ cells/well) were incubated in 96-well plates in DMEM for 24 h. Then, HepG2 cells were treated with different concentrations of TIIA (0, 1.25, 2.5, 5, 10, and 20 μM) or adriamycin (ADR) (0, 0.5, 1, 2, 4, and 8 μM) for 48 h. The cell viability was measured by MTT assay. Then, 20 μL MTT was added to each well and incubated for 3.5 h. The supernatant was discarded, and 150 µL of DMSO was used to dissolve the formazan crystals. The samples were placed into the shaker for 10 min, to fully dissolve the purple formazan. Their absorbance was measured with a microplate reader at 490 nm. ADR was used as a positive control and the reference drug in this experiment. Cell viability (%) = OD_sample_/OD_control_ × 100%. The cell viability of TIIA was performed in triplicate and expressed as the half-maximal inhibition concentration (IC_50_) value.

L02 cells (1 × 10^4^ cells/well) were incubated in 96-well plates in 1640 for 24 h. Then, the cells were treated with different concentrations of tanshinone IIA (0, 2.5, 5, 10, 20, and 40 μM) for 48 h to detect the cytotoxicity. The remaining steps were the same as before.

### 4.5. Cell Cloning

HepG2 cells (600 cells/well) were incubated in 6-well plates and were treated with TIIA (2.5 and 10 μM) or ADR 2 μM. The culture medium was changed every 3 days; 7 days later, visible colonies had formed. Then, the cells were washed twice with PBS, and the colonies were fixed with 4% paraformaldehyde for 15 min; the fixative was removed, and they were stained with 600 μL crystal violet for 15 min. The staining solution was washed away, and then the colonies were dried and then photographed.

### 4.6. Scratch and Transwell Testing

HepG2 cells (3 × 10^5^ cells/well) were incubated in 12-well plates. After 24 h, the cells were grown to full confluence in plates and scratched using a 200 μL pipette tip. The cells were washed with PBS to remove cellular debris. Then, the cells were treated with TIIA (2.5 and 10 μM) or ADR 2 μM for 48 and 72 h. Scratch healing rate = [(0 h_scratch width_ − 36/72 h_scratch width_)/0 h_scratch width_] × 100%.

HepG2 cells (8 × 10^5^ cells/well) were incubated in Transwell inserts in an FBS-free DMEM. The control group was incubated in DMEM medium with 10% FBS in a 5% CO_2_ chamber at 37 °C for 48 h. The culture medium was discarded, and the cells were fixed with 4% paraformaldehyde for 20 min and stained with 0.1% crystal violet for 30 min. Then, they were photographed to observe cell migration. 

### 4.7. Annexin V/PI Stain

HepG2 cells (8 × 10^5^ cells/well) were incubated in 6-well plates for 24 h. Then, cells were treated with 2.5 and 10 μM of TIIA or ADR 2 μM for 24 h. Detached cells were collected by centrifugation (1200 rpm/min, 5 min) and attached cells by trypsinization. They were washed twice with PBS, and the cells were gently resuspended using 400 μL Annexin V-FITC binding solution; then, 5 μL Annexin V-FITC and 5 μL propidium iodide (PI) were added, and the solution was incubated in the dark for 10 min. Subsequently, the samples were processed and analyzed using flow cytometry.

### 4.8. Real-Time Quantitative PCR Analysis

L02 and HepG2 cells (8 × 10^5^/well) were incubated for 24 h. Then, the L02 and HepG2 cells were collected; total mRNA was extracted using Trizol reagent. Reverse transcription was performed using the ReverTra Ace qPCR RT kit. According to the manufacturer’s protocol, Q-PCR was performed in a real-time PCR system. The primer sequences were synthesized by Sangon Biotech (Shanghai, China) in Table 1. Relative mRNA expression was computed using the 2^−ΔΔ^Ct method.

### 4.9. Western Blot

Tumor tissues or HepG2 cells were lysed in RIPA lysis buffer for 30 min at 4 °C, and lysates were collected by centrifugation at 12,000 rpm for 10 min. The protein samples were separated through 4–12% SDS-PAGE and then transferred to PVDF membranes. The PVDF membrane was incubated with 5% skim milk for 2 h. Subsequently, the primary antibodies were incubated at 4 °C overnight. The membranes were washed with PBST three times, 10 min/time. The secondary antibody was incubated at room temperature for 1 h. Finally, the protein bands were detected using the ECL method and analyzed with Image J software 1.53 t. 

### 4.10. Fluorescence Staining1

HepG2 cells (4 × 10^5^/well) were incubated for 24 h and then treated with the drug for 24 h. The cells were washed 3 times with PBS, at room temperature; then, 1 mL of 4% paraformaldehyde was added to each well for 60 min and, finally, the cells were washed 5 times with PBS. After blocking with an antibody-blocking solution (FDB) for 30 min, the cells were incubated with the primary antibody (1:100 dilution) overnight at 4 °C and washed 5 times with PBS, and 200 μL of the secondary antibody (FITC-goat anti-rabbit IgG antibody, 1:200-fold dilution) was added to each well and left for 60 min. The cells were washed 5 times with PBS, incubated with DPAI (100 μL/well) for 7 min at room temperature in the dark, and then washed 5 times with PBS. The cells were detected with a laser confocal microscope.

### 4.11. Reactive Oxygen Species Assay

HepG2 cells (4 × 10^5^/well) were incubated for 24 h and then treated with the drug for 24 h. The DCFH-DA (1:1000-fold dilution) of 1mL/well was added to each well and incubation was continued for 20 min. The cells were washed 3 times with DMEM. The cells were detected using a fluorescence microscope.

### 4.12. Molecular Docking

The structure of PERK (PDB code 4G34) was obtained from RCSBPDB (https://www.rcsb.org/, accessed on 14 December 2023) as a receptor. The three-dimensional chemical structure of ginsenoside TIIA was obtained from Pubchem (https://pubchem.ncbi.nlm.nih.gov/, accessed on 14 December 2023), and both were prepared for ligand binding. These were preserved in the PDB format for the following procedures. By optimizing the AutoDock4 software (1.5.6 Sep_17_14), TIIA interacted with PERK at a molecular simulative level. The docking center was set as center_x 41.173, center_y −18.656, and center_z 3.886, respectively, and the number of individual points was set as 60.

### 4.13. Cellular Thermal Shift Assay

The total protein of HepG2 cells was obtained using RIPA lysis buffer for 30 min at 4 °C, and lysates were collected by centrifugation at 12,000 rpm for 10 min. The protein samples were used with or without TIIA, and then we incubated the TIIA at 4 °C for 1 h. Cells were subpackaged into 12 EP tubes (50 μL/each) and heated with a thermal gradient from 40 to 95 °C for 5 min. A 20 μL aliquot of the supernatant was loaded onto an SDS-PAGE gel, followed by Western blotting. Cellular Thermal Shift Assay (CETSA) curve analysis was conducted using GraphPad Prism 8 software.

### 4.14. Enzyme-Linked Immunosorbent Assay 

HepG2 cells (2.5 × 10^6^ cells/well) were incubated in 6-well plates for 24 h and were treated with TIIA (2.5 and 10 μM) or ADR 2 μM. According to the instructions supplied by the manufacturer, 1 × 10^6^ cells were added to 0.2 mL of reagent 1 for 10 min at 4 °C, and then the supernatant was collected by centrifugation (12,000 rpm/min, 5 min). Subsequently, the relevant reagents were added to evaluate the relative Fe^2+^ content in HepG2 cells.

### 4.15. Glutathione Assay Kit

Fresh tumor tissue was washed twice with PBS, and then 100 mg of tumor tissue was weighed and placed in a 2 mL grinding tube. According to the instructions supplied by the manufacturer, 1 mL of reagent was added to the grinding tube and thoroughly ground in a homogenizer, and the supernatant was collected by centrifugation at 12,000 rpm for 10 min at 4 °C. Then, the relevant reagents were added to evaluate the relative glutathione (GSH) content in the tumor tissue.

### 4.16. H&E Staining

Fresh tumor tissues were fixed in 4% paraformaldehyde. Then, the tissues were dehydrated, processed, and embedded in paraffin. Serial sections with 5 µm thickness were prepared and stained with hematoxylin and eosin (H&E) for histopathological evaluation using a light microscope.

### 4.17. Immunohistochemical Analysis

The tissue sections were deparaffinized and rehydrated with xylene and anhydrous ethanol, respectively. After antigen retrieval in citrate buffer solution (0.01 M, pH 6.0) at room temperature for 10 min, the sections were washed three times with PBS and incubated with primary antibodies for 1 h, followed by a secondary antibody at room temperature for 20 min. After DAB staining for 8 min and hematoxylin counterstaining for 20 s, the sections were dehydrated in ethanol.

### 4.18. Statistics

The statistics were analyzed using SPSS 26.0 software. Data are presented as the mean ± standard deviation (SD). Multiple group comparisons were analyzed with one-way ANOVA, and pairwise comparisons were analyzed using SNK. If the data did not conform to normality, we performed nonparametric testing. *p* < 0.05 was considered statistically significant.

## 5. Conclusions

**Figure 9:**
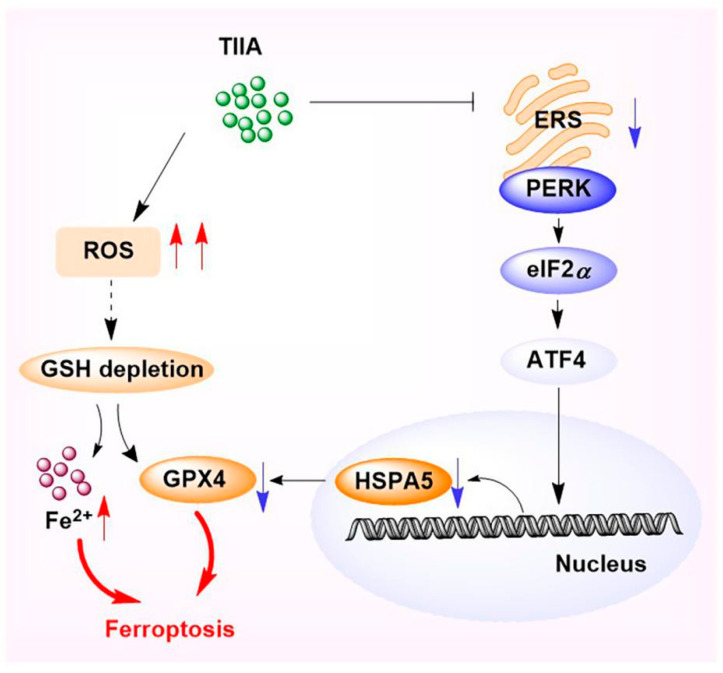
The possible anticancer mechanism of TIIA.

Taken together, the results of this study suggest that ferroptosis is a novel anticancer mechanism of TIIA in HCC, in which TIIA induces ferroptosis in HCC cells by inhibiting the PERK-ATF4-HSPA5-GPX4 signaling pathway. The possible mechanism is shown in Figure 9.

## Figures and Tables

**Table 1:** Primer sequences for qPCR.

Target Gene	Primer Sequences (5′-3′)
PERK (forward)	GATCGCAGAGGCAGTGGAGTTTC
PERK (reverse)	TTGTCCTGTGTGTCTGGCATAAGC
GAPDH (forward)	TTGCCCTCAACGACCACTTT
GAPDH (reverse)	TGGTCCAGGGGTCTTACTCC

## Data Availability

Data are contained within the article.
